# Microstate Changes Associated With Alzheimer’s Disease in Persons With Down Syndrome

**DOI:** 10.3389/fnins.2019.01251

**Published:** 2019-11-28

**Authors:** Christian Sandøe Musaeus, Lise Cronberg Salem, Troels Wesenberg Kjaer, Gunhild Waldemar

**Affiliations:** ^1^Danish Dementia Research Centre, Department of Neurology, Rigshospitalet, University of Copenhagen, Copenhagen, Denmark; ^2^Department of Clinical Medicine, University of Copenhagen, Copenhagen, Denmark; ^3^Neurophysiology Center, Zealand University Hospital, Roskilde, Denmark

**Keywords:** Down syndrome, EEG, Alzheimer’s disease, microstates, diagnostic

## Abstract

Down syndrome (DS) is associated with development of dementia due to Alzheimer’s disease (AD). However, due to considerable heterogeneity in intellectual function among persons with DS, it is difficult to assess whether a person with DS has developed dementia due to AD (DS-AD). EEG spectral power has previously shown very promising results with increased slowing in DS-AD compared to DS. However, another technique called microstates may be used to assess whole-brain dynamics and has to our knowledge not previously been investigated in either DS or DS-AD. The aim of the current study was to assess whether microstates could be used to differentiate between adults with DS, and DS-AD. We included EEGs from 10 persons with DS and 15 persons with DS-AD in the analysis. For the microstate analyses, we calculated four global maps, which were then back-fitted to all the EEGs. Lastly, we extracted the duration, occurrence, and coverage for each of the microstates. Here, we found the four archetypical maps as has previously been reported in the literature. We did not find any significant difference between DS and DS-AD but the largest difference in microstate duration between DS and DS-AD was found in microstate A and D. These findings are in line with structural MR studies showing that both the frontal and temporal lobes are affected in persons with DS-AD. Microstates may potentially serve as a diagnostic marker, but larger studies are needed to confirm these findings.

## Introduction

Down syndrome (DS) is the most common chromosomal defect, which leads to mental retardation and is caused by trisomy of chromosome 21 (Bittles et al., 2007). Studies have found that DS is associated with later development of dementia due to Alzheimer’s disease (AD), and that the neuropathological features of AD are present in adults and even children with DS (Olson and Shaw, 1969; Mann, 1988; Zigman and Lott, 2007; Wilcock and Griffin, 2013). However, the number of persons with DS who develop AD (DS-AD) varies between studies with one study showing that 9% of adults with DS in their thirties and around 55% in their fifties suffer from dementia (Prasher and Filer, 1995). Another study has found that nearly 20% of people with DS at the age of 45 or more suffers from AD (McCarron et al., 2014).

One reason for varying estimates may be that persons with DS have a low baseline intellectual function with considerable heterogeneity, which makes it difficult to establish reliable cut-off scores on cognitive tests. Furthermore, AD has an initial presentation in DS that differs compared to AD in the general populations, often with changes in personality and executive function seen before memory impairment (Wilson et al., 2014). This may be due to abnormalities in the brain development that results in hypoplasia of the frontal lobes in persons with DS, and thus vulnerability to amyloid depositions (Holland et al., 2000). In addition, persons with DS may struggle with communication which leads to a reliance on informant-based questionnaires when documenting cognitive impairment. Ideally, the clinical evaluation should be supplemented by biomarkers. Such biomarkers could include atrophy on structural scans such as CT or MRI. However, due to their intellectual disability it is often difficult for persons with DS to lie still when performing MRI scans and thereby the assessment of brain networks with fMRI can be difficult.

Electroencephalography (EEG) is on the other hand easier to apply and methods like EEG-based microstate analysis have been able to show topographical maps associated with resting state networks as measured with resting state fMRI (Van de Ville et al., 2010; Yuan et al., 2012). Microstates is a technique where the EEG signals are divided into a number of distinct states (Lehmann et al., 1987). The states occur in a time range of milliseconds but it has been shown that momentary stable spatial patterns occur, which last approximately 100 ms (Khanna et al., 2015). Studies looking at the clinical applicability of EEG microstates have found alterations in the structure and temporal representation of microstates in both AD (Ihl et al., 1993; Dierks et al., 1997; Strik et al., 1997; Stevens and Kircher, 1998; Nishida et al., 2013; Musaeus et al., 2019a), frontotemporal dementia (Nishida et al., 2013) and schizophrenia (Lehmann et al., 2005; Irisawa et al., 2006; Kikuchi et al., 2007; Kindler et al., 2011; Nishida et al., 2013; Andreou et al., 2014; Tomescu et al., 2014) thereby supporting it as a novel biomarker of both neurological and psychiatric disease. Meanwhile, no studies have so far investigated this technique in persons with DS or whether it could be used to assess whether a person with DS has developed AD.

In the current exploratory study, we assessed whether microstates could be used to differentiate between persons with DS and persons with DS-AD. In addition, we wanted to explore whether the scores on an informant-based questionnaire were associated with the changes in the microstates that are related to frontal and temporal brain areas.

## Materials and Methods

### Participants

The persons with DS-AD were recruited from the Memory Clinic at Rigshospitalet while the adults with DS were recruited from institutions for adults with intellectual disabilities. Informed consent was obtained from the legal guardian, or if no legal guardian was appointed, the family doctor gave informed consent. Both the subjects and caregivers were informed that they could request the interruption of the clinical procedures at any time. This study was approved by the Regional Ethical Committee.

### Inclusion and Exclusion Criteria

The following inclusion criteria were applied for persons with DS-AD: (1) karyotype examination, which confirm trisomy of chromosome 21; (2) ability to cooperate; (3) over 35 years old, and (4) fulfilling the clinical criteria for probable AD (McKhann et al., 1984). The inclusion criteria for the DS were the fulfillment of criteria 1–3 and lack of fulfillment of criteria 4. The exclusion criterion for all participants was an untreated somatic or psychiatric condition that may influence cognition.

A total of 21 persons with DS-AD and 16 with DS and no cognitive decline were recruited as assessed with the informant-based Dementia Screening Questionnaire in Intellectual Disability (DSQIID) (Deb et al., 2007), which has been shown to be a valid and reliable observer-rated questionnaire for screening for dementia among adults with DS (Deb et al., 2007; Gomiero et al., 2017).

### Clinical Assessment

We performed a medical history including medication status and history of symptoms of dementia from family members and/or caregivers to establish the dementia diagnosis. Furthermore, the participants were assessed with physical and neurological examinations including assessment of symptoms of depression. Furthermore, we used the informant-based questionnaire DSQIID to screen for dementia symptoms. Lastly, blood tests were performed in accordance with international clinical guidelines available (Hort et al., 2010) and confirmation of genetic status from the medical records were investigated. Furthermore, if the person could cooperate, cranial CT was performed.

Dementia was diagnosed according to the ICD-10 and/or DSM-IV criteria. The diagnosis of AD was established according to the criteria of the NINCDS-ADRDA criteria for probable AD (McKhann et al., 1984), supported by the ICD-10 Symptom Checklist for Mental Disorders and international guidelines by the International Association for the Scientific Study of Intellectual Disabilities (Aylward et al., 1997). A consensus diagnosis was established by a multidisciplinary team after the initial work-up. The severity of the dementia was found to be in a mild-to-moderate phase. Each control subject with DS included in this study was examined at baseline with all procedures except CT.

### Electroencephalography Recording

The participants were instructed to lay on a bed and try to relax and if it was possible then asked to close their eyes. If needed, the technician was holding the participants hands. The EEGs were recorded using Nicolet One EEG (Nervus) recording software 5.82 (Natus) with a standard 44-channel headbox. Each subject was fitted with a cap using silver-silver-chloride-coated electrodes and the data were sampled at 1 kHz. The EEGs was recorded in a 30-min period in subjects in the wake resting state from 19 electrodes positioned according to the International 10–20 system. For impedance, the aim was to reach below 10 kOhm for all electrodes during the recordings. However, we do not have any records of the impedance before or after the recording.

### Preprocessing of Electroencephalography

Results from analysis of spectral power have been presented elsewhere (Salem et al., 2015; Musaeus et al., 2019b). All preprocessing was performed in MATLAB (Mathworks, v2016a) using the EEGLAB toolbox (Delorme and Makeig, 2004). The electrodes were computationally located on the scalp using the DIPFIT toolbox (Oostenveld et al., 2011) with the standard 10–20 cap model. The excessive channels were removed, and the data was bandpass filtered from 1 to 70 Hz, and bandstop filtered from 45 to 55 Hz using the *pop_firws* function in MATLAB, with a filter order of 2. Afterward, the data were then re-referenced to a common average montage. Then segments of both eyes open and closed were selected. Next, the data was divided into 1-s epochs and the EEGs were visually inspected and epochs with excessive artifacts were removed. If there was less than three channels with excessive artifacts, they were then interpolated using spherical interpolation otherwise the segments were rejected. Afterward, independent component analysis was performed with the extended infomax algorithm (Lee et al., 1999), and components containing eye blinks or eye movement were removed. Lastly, the EEGs were visually inspected and epochs with artifacts were removed. The person performing the preprocessing was blinded to whether the participants were DS or DS-AD.

After the preprocessing, only subjects with at least 30 1-s epochs were used for further analysis. Furthermore, all epochs were selected within the first 30 s after the participants closed their eyes to avoid any effects from drowsiness or sleep. In the eyes closed condition, EEGs from 16 DS-AD, and 12 DS were included. We did not look at the eyes open segments due to the varying activities and focus for the participant when they had their eyes open.

### Microstate Analysis

The microstates analysis was performed using the Microstate EEGlab Toolbox (Poulsen et al., 2018). Before the microstate analysis, we first band-pass filtered the data between 2 and 20 Hz with the same settings as mentioned above. Afterward, we concatenated the epochs for each subject, i.e., ending up having one continuous EEG file instead of 1-s epochs. To assure the quality of the individual microstate maps, we first extracted the global field power (GFP) peaks for each participant with the following settings: minimum peak distance of 10 ms, the number of GFP peaks was set at the maximum for the shortest EEG file, and GFP peaks that exceeded two times the standard deviation of the GFP of all maps were excluded. For segmentation, we used the Topographic Atomize and Agglomerate Hierarchical Clustering (TAAHC) algorithm. Afterward, each map was visually inspected and subsequently removed from the analysis if they did not resemble the four maps previous reported in the literature (Michel and Koenig, 2018). Here, we excluded EEGs from two persons with DS, and one person with DS-AD.

**FIGURE 1:**
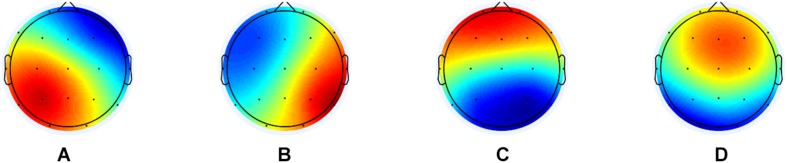
The global maps that were calculated based the aggregated dataset from all participants and were back-fitted to each of the EEG recordings. The labels **(A–D)** are according to the previous literature in the microstate field.

In the final analysis, we concatenated the GFP peaks from all subjects (n_*DS*_ = 10, n_*DS–AD*_ = 15) into one file before segmentation. This was done for the maximum number of peaks for the shortest EEG file (GFP peaks = 508) with the goal to maximize the similarity between the microstates they would be assigned to, and not to make the contribution to the global maps uneven between groups. For segmentation, we used the TAAHC algorithm with the same settings as described above. First, we estimated four microstates, since that has been reported as the most common (Khanna et al., 2014) and reproduceable (Khanna et al., 2015). Due to the low GEV, we also extracted both five and six microstates (see Supplementary Material). The global maps (see Figure 1) were then back-fitted to each of the EEG files by labeling each of EEG segments with the class of microstates it is most familiar. The labels A-D are accordance to the previous literature in the microstate field. The labels of time frames in small segments (less than 30 ms) were changed to the next most likely microstate class, as measured by global map dissimilarity (Poulsen et al., 2018). After back-fitting the global maps, we calculated global explained variance (GEV), duration, occurrence, and coverage for the EEG files.

Duration was defined as the average time for each map to be present before transitioning to another map while occurrence is defined as the average of times a microstate occurred during each second, and coverage is defined as the percent of the EEG recording that a microstate was accounted for. GEV is defined as the variance of EEG activity explained by all four microstates.

For the syntax analyses, we performed the same analysis as previously described (Lehmann et al., 2005; Nishida et al., 2013). Basically, we calculated the observed transitions based on all transitions and then the expected transitions based on the occurrence of the microstates for each subject separately. The values were averaged across subjects for each group, and the difference was assessed using the chi-square distance. We performed a permutation test with 5000 repetitions where the labels ‘expected’ and ‘observed’ were randomly assigned to the subjects’ sets of the 12 transition probabilities, and the chi-square distance was computed.

### Statistical Analysis

All statistical analyses were performed in Mathworks, v2016a. Firstly, we compared age and DSQIID score between DS, and DS-AD using Wilcoxon rank sum test. When comparing DS-AD and DS we performed an ANCOVA with age and gender as covariates for each of the microstate features with the significance level set at 0.05. Before performing the ANCOVA, we log-transformed the data. Due to the exploratory nature of the study, we did not correct for multiple comparisons. To assess whether the scores were associated with clinical measures, we performed correlation using Spearman’s rho between DSQIID and the duration of microstate D and the duration of microstate A. We choose to correlate the microstate features with the DSQIID score since it represented a broad number of areas.

## Results

### Demographics

The mean age was lower in the DS group [mean (SD) = 47.1 (9.49)] as compared to the DS-AD group [mean (SD) = 51.80 (5.13)] with a *p*-value of 0.055. However, no differences in gender was found between DS and DS-AD with a *p*-value of 0.137. The mean DSQIID score was significantly higher in the DS-AD [mean (SD) = 21.6 (5.72)] compared to DS [mean (SD) = 2.60 (2.84)] with a *p*-value of < 0.001. The number of 1-s epochs for DS-AD [mean (SD) = 86.80 (52.85)] was not significantly different from DS [mean (SD) 108.3 (57.72)] with a *p*-value of 0.347.

### Microstate Features

See Figure 1 for global maps of the microstates that were used for back-fitting and Table 1 for the mean values, standard deviation, and *p*-values. The average GEV was not significantly different between DS (mean = 58.99%, *SD* = 4.02), and DS-AD (mean = 58.92%, *SD* = 7.96) (*p*-value = 0.979). When examining the microstate features, we found a shorter duration for microstate D and a longer duration for microstate A for DS-AD as compared with DS, see Table 1. The largest difference was found for microstate A (*p*-value = 0.091, *t*-value = 3.131). The same pattern was found for occurrence, and coverage for microstates A and D. See Supplementary Material for results from five and six microstates. When extracting six microstates, we found that there was a larger difference in the duration of microstate D1, which is more centered on the left side as compared with D2, which is more centered on the right side (see Supplementary Figure 2).

**TABLE 1:** Mean, standard deviation (SD), and *p*-value for comparisons between DS and DS-AD for microstates A-D for duration, occurrence, and coverage.

	**Duration**				**Occurence**				**Coverage (%)**			
	**A**	**B**	**C**	**D**	**A**	**B**	**C**	**D**	**A**	**B**	**C**	**D**
**DS**	65.94 (6.06)	70.79 (8.77)	91.61 (19.72)	81.52 (14.55)	2.75 (0.54)	2.74 (0.33)	3.66 (0.52)	3.37 (0.58)	18.31 (4.81)	19.63 (4.63)	33.98 (10.33)	28.08 (9.30)
**DS-AD**	73.19 (7.80)	71.08 (6.66)	89.77 (18.22)	75.79 (10.73)	3.03 (0.58)	2.88 (0.58)	3.59 (0.47)	3.19 (0.54)	22.45 (6.20)	20.69 (5.24)	32.39 (7.99)	24.47 (6.94)
***p*-Value**	0.091	0.740	0.553	0.342	0.359	0.218	0.988	0.834	0.197	0.320	0.698	0.548

*DS, Down syndrome; DS-AD, Down syndrome with Alzheimer’s disease.*

No significant differences were found for the syntax analyses.

### Correlations

**FIGURE 2:**
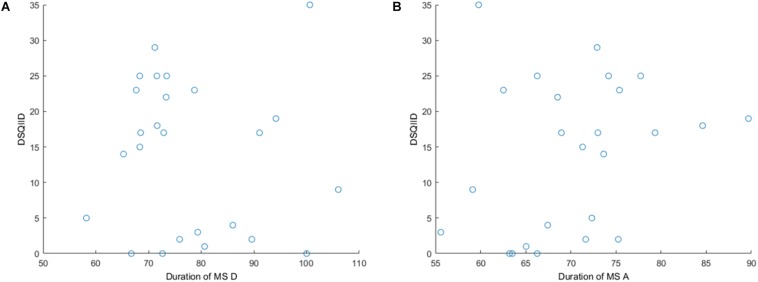
Scatterplots for **(A)** DSQIID and the duration of microstate D, and **(B)** DSQIID and the duration of microstate A.

We performed the Spearman’s correlation between DSQIID and the duration of microstate A and found a negative correlation (*p*-value = 0.617, ρ = −0.105). Furthermore, we correlated the duration of microstate A with the DSQIID score and found a positive correlation (*p*-value = 0.128, ρ = 0.313). See Figure 2 for scatterplot.

## Discussion

In the current exploratory study, we found that the largest differences in microstate features were found for microstate D and in particular for microstate A in persons with DS compared to DS-AD. Specifically, DS-AD had a shorter duration of microstate D, and a longer duration of microstate A as compared to DS. No significant differences were found for the syntax analyses. Lastly, we found that the DSQIID score was positively correlated with the occurrence of microstate A (*p*-value = 0.128, ρ = 0.313) although not significant.

No previous studies have to our knowledge investigated the microstate changes in persons with DS-AD nor in persons with DS. However, previous studies have investigated microstates in patients with AD (Ihl et al., 1993; Dierks et al., 1997; Strik et al., 1997; Stevens and Kircher, 1998; Nishida et al., 2013) without DS and the majority found a shorter duration of the microstates in patients suffering from AD (Dierks et al., 1997; Strik et al., 1997; Stevens and Kircher, 1998) compared to healthy older controls. One recent study did not find any significant difference between patients with AD and healthy controls (Nishida et al., 2013), which could be due to low sample size or as previously suggested temporal disorganization in patients with AD (Koenig et al., 2005; Nishida et al., 2013). Another study found that patients with AD showed an increased occurrence of microstate A compared to healthy controls (Musaeus et al., 2019a). In the current study, we found that features of microstate A were indeed increased in DS-AD compared to DS when applying four microstates, which is in line with one of the more recent studies (Musaeus et al., 2019a). This finding could suggest that microstate A is associated with underlying AD pathology in the temporal lobes. However, we also found a large difference in duration in microstate D. This difference may be more pronounced in the left frontal lobe since D1 was more affected than D2 as seen in Supplementary Figure 2 and Supplementary Table 2. This difference may be due to the initial presentation of AD in DS with changes in personality and executive function before memory impairment (Wilson et al., 2014; Fonseca et al., 2016). This is further supported by recent imaging studies using MR (Powell et al., 2014; Sabbagh et al., 2015) showing that also the frontal areas of the brain are affected in persons with DS-AD. No significant correlations were found between DSQIID and the duration of microstate A, and D. This may in large part be due to the DSQIID examining multiple domains. Overall, these findings support the notion that the symptoms of AD in DS are due to affection of both temporal and frontal areas of the brain. However, larger studies are needed to confirm these findings.

Microstate classes have also been associated with BOLD signal and resting state networks obtained with fMRI in multiple studies (Britz et al., 2010; Van de Ville et al., 2010; Yuan et al., 2012). Here, microstate A has been associated with the superior and middle temporal gyri as well as the left middle frontal gyrus (Britz et al., 2010). Furthermore, microstate D has been associated with BOLD activations in the right superior and middle frontal gyri as well as the right superior and inferior parietal lobules (Britz et al., 2010). Both the temporal and frontal areas of the brain have been associated with the early development of AD as measured with beta-amyloid depositions using PiB-PET in persons with DS (Landt et al., 2011; Sabbagh et al., 2011; Handen et al., 2012; Jennings et al., 2015). The reason for the frontal affection may be explained by the underdevelopment of the frontal lobe in persons with DS, which may make it more vulnerable to amyloid depositions (Holland et al., 2000). However, longitudinal studies are needed to assess the order of microstate changes during the development of AD in persons with DS.

Looking at the GEV, we found it was not significantly different between the groups for any of the analyses but was low compared to other studies with most commonly reporting a GEV > 70% (Michel and Koenig, 2018). There may be multiple reasons for the lower GEV in the current study. First, we extracted global maps from the GFP peaks of all the subjects to create global maps as opposed to extracting maps for each subject. By doing this, we decreased the GEV. Secondly, the filter width was 2–20 Hz, and it may increase the GEV to narrow the band width since microstates are mostly based on the alpha band. In an attempt to examine whether increasing the number of microstates would increase the GEV, we also looked at both five and six microstates (see Supplementary Material) but this only increased the GEV by a few percents (five microstates = 60.00%, six microstates = 61.39%). In the current analysis, we included only the maximum number of GFP peaks for the shortest EEG to the segmentation to avoid problems in terms of larger contributions from the longer EEG files in creating the global maps.

The current study has some limitations. Firstly, there was a difference in the age between the DS, and DS-AD, which is due to the prevalence of AD in persons with DS increases with age (Head et al., 2012). In an attempt to overcome this issue, we have used both age and gender as covariates in the ANCOVA. In addition, the sample size is small, making it hard to set up reliable classification models, and as previously mentioned, larger studies are needed to confirm the findings from the current study. However, we demonstrated changes in microstates in general and specifically in microstate A and D reflecting temporal and frontal network changes in persons with DS developing AD. Secondly, individuals with a severe intellectual disability were excluded from the study, and therefore results may not be generalizable to all individuals with DS. Lastly, some studies have recorded EEG with clear instructions to the participants when they should close and open their eyes. This is not the case in the current study, it is therefore possible that drowsiness is a potential confounder of the data. However, structured EEG recordings might be difficult as adults with intellectual impairment can have difficulty cooperating.

## Conclusion

In the current study, we found that the microstates associated with temporal and frontal areas of the brain were altered in DS-AD compared to DS. These findings suggest that the initial functional brain changes in persons with DS, who develop AD, are both temporal and frontal. This is in line with some studies showing that affected executive function may be an initial symptom in person with DS-AD and imaging studies showing that DS-AD display both temporal and frontal atrophy. EEG microstates may potentially serve as a diagnostic marker, but larger studies are needed to confirm these findings.

## Data Availability Statement

The datasets supporting the conclusions of this manuscript will be made available by the authors to any qualified researcher. However, due to regulations, we are not able to share the EEG files.

## Ethics Statement

The studies involving human participants were reviewed and approved by the Regional Committee on Health Research Ethics. Written informed consent was obtained from the legal guardian, or if no legal guardian was appointed, the family doctor gave written informed consent.

## Author Contributions

CM, LS, TK, and GW conceived the project idea of using quantitative EEG and contributed to revising the manuscript. LS conducted the experiments. CM conducted the data analyses and drafted the manuscript.

## Conflict of Interest

The authors declare that the research was conducted in the absence of any commercial or financial relationships that could be construed as a potential conflict of interest.
