# Supplementary material for: Microstate Changes Associated With Alzheimer’s Disease in Persons With Down Syndrome
Source: Front Neurosci. 2019 Nov 28;13:1251. doi: 10.3389/fnins.2019.01251 (PMC6892825; doi:10.3389/fnins.2019.01251)
Supplement: Supplementary file 1 [file Table_1.DOCX]

**Supplementary material**


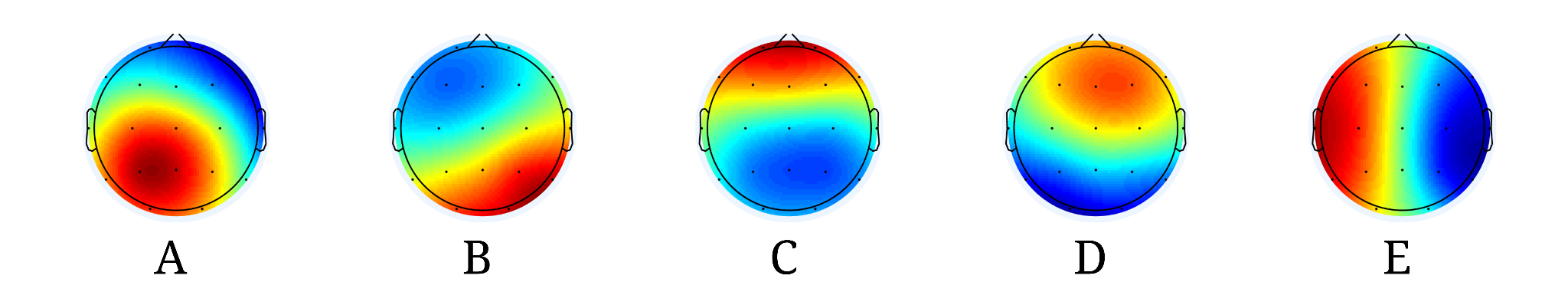


**Supplementary figure 1**: The global maps calculated based on the aggregated dataset from all participants and back-fitted to each of the EEG recordings.

**Supplementary table 1**: Table showing the mean, standard deviation (SD), and p-value for comparisons between DS and DS-AD when extracting five microstates for microstates A-E for duration, occurrence, and coverage. DS = Down syndrome, DS-AD = Down syndrome with Alzheimer’s disease.


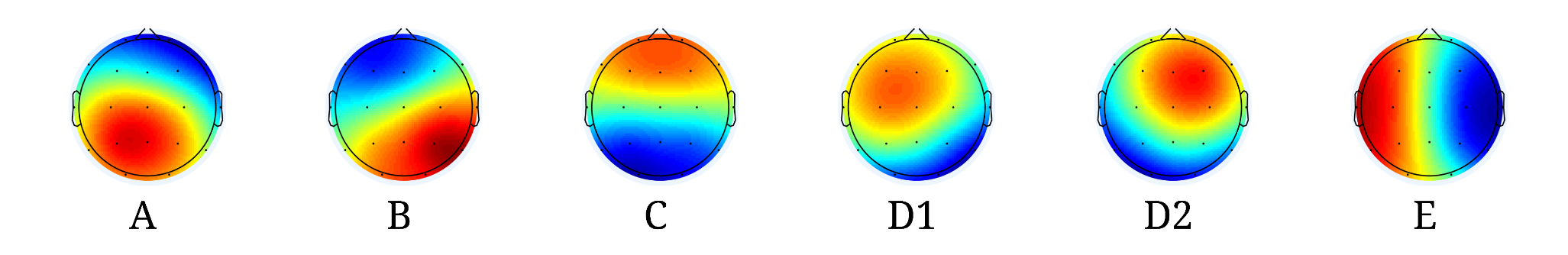


**Supplementary figure 2**: Global maps calculated based on the aggregated dataset from all participants and back-fitted to each of the EEG recordings.

**Supplementary table 2**: Table showing the mean, standard deviation (SD), and p-value for comparisons between DS and DS-AD when extracting six microstates for microstates A-E for duration, occurrence, and coverage. DS = Down syndrome, DS-AD = Down syndrome with Alzheimer’s disease.
